# The Prevalence and Risk Factors for Low Birth Weight among Term Newborns in Adwa General Hospital, Northern Ethiopia

**DOI:** 10.1155/2017/2149156

**Published:** 2017-07-04

**Authors:** Yisak Gebregzabiherher, Abera Haftu, Solomon Weldemariam, Haftom Gebrehiwet

**Affiliations:** ^1^Midwifery Department, Adwa General Hospital, Adwa, Ethiopia; ^2^Midwifery Department, Mekelle University, Mekelle, Ethiopia

## Abstract

*Background. *World health organization estimates that 25 million LBW babies are born annually worldwide and 95% occur in developing countries.* Objective*. To assess the prevalence and associated factors of low birth weight among term neonates delivered in Adwa Hospital, Northern Ethiopia.* Methods*. A cross-sectional study was conducted among neonates delivered in Adwa Hospital. All live births delivered from July 1, 2014, to June 30, 2016, were included in this study. The study participants were selected through systematic sampling technique and the data was collected using a structured questionnaire. Data was entered to Epi Data version 3.1 and analyzed using SPSS version 20 software. To identify independent predictors, bivariate and multivariable binary logistic regressions were employed. Adjusted odds ratio and 95% confidence interval were used to determine the strength of association.* Results. *The prevalence of term low birth weight was 10%. The risk factors were mothers aged less than 20 years, mothers whose pregnancy was desired, mothers with a history of abortion, and mothers with normal hemoglobin, iron with folic acid, and HIV status.* Conclusion. *The burden of LBW obtained in this study was in the same range as in some other countries.

## 1. Introduction

Birth weight is the first weight of the foetus or newborn obtained after birth. Low birth weight refers to birth weights below 2500 grams [1]. It is one of the major determinants of perinatal survival, infant morbidity, and mortality as well as the risk of developmental disabilities and illnesses in future [2]. Neonatal death among infants weighing 1500–2500 grams is 20 times higher than among infants of normal weight [3, 4]. WHO estimated that about 25 million low birth weight babies are born each year, nearly 95% of them in developing countries [5].

More than 20 million infants worldwide representing 15.5% of all births are born with low birth weight, 95.6% of them in developing countries [6, 7]. The prevalence of low birth weight in developing countries (16.5%) is twice that in developed regions (7%) [8]. A study done in Gondar showed that the overall prevalence of low birth weight was 17.4% [9]. LBW is considered as the single most important predictor of infant mortality, especially of deaths within the first months of life [10]. It is also a significant determinant of infant and childhood morbidity, particularly of neurodevelopment impairment such as mental retardation and learning disability [11]. Half of all perinatal and one-third of all infant deaths are directly or indirectly related to LBW [12]. Mortality of LBW babies is 40 times more than the normal weight babies [13]. Infants born with very low weight are more than 100 times more likely to die in the first year of life than are infants of normal birth weight [14]. The objective of this study was to assess the prevalence and associated factor of low birth weight in Adwa Hospital, North Ethiopia.

**Figure 1:**
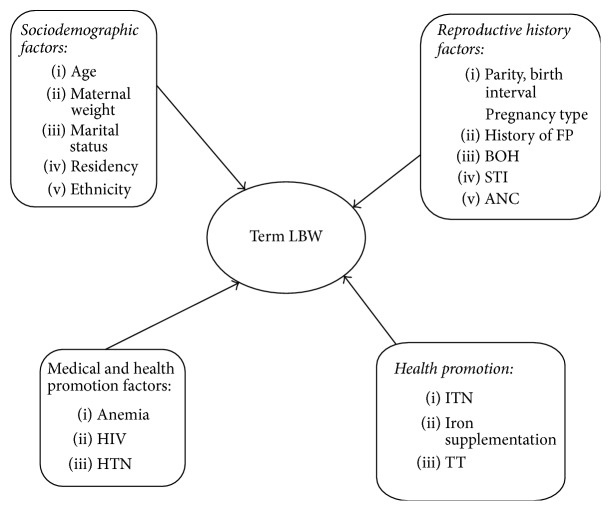
Conceptual framework to assess the prevalence and its associated factors of low birth weight among term neonates delivered in Adwa General Hospital, Northern Ethiopia.

As far as our knowledge, studies done on LBW and its risk factors are limited in Ethiopia. Therefore, the aim of this study is to assess prevalence of TLBW and its risk factors in Tigray region (Figure 1). This study might contribute by providing pertinent information for policy makers and health system planers for possible modifications of strategies to reduce TLBW. It is also important for clinicians who are working in maternity area to adjust and give emphasis in giving care during ANC.

## 2. Subjects and Methods

Adwa city is located in central zone of Tigray with an area of 23.0316 km^2^ and total population of 66826. It is 1004 km far from the capital city of Ethiopia, Addis Ababa, and 223 km from Mekelle, which is a capital city of Tigray. The study was conducted between July 1, 2014, and June 30, 2016. Cross-sectional study design was conducted at Adwa Hospital to collect the data.

The study population was sampled term newborn babies delivered at Adwa General Hospital for the period July 1, 2014, to June 30, 2016. Individual term newborn babies were reviewed from mother's chart or cards.

*Inclusion Criteria*. Incorporated babies of all pregnant mothers delivered between 37 up to 42 week gestational age and singleton births were included.

*Exclusion Criteria*. Babies born to mothers with medically known diabetes, babies born to mothers with hypertension, babies born to mothers with sickle cell hemoglobinopathy, records with incomplete data, postterm, preterm, and stillbirth were excluded. Sample size was calculated by using single population proportion formula (*n* = (*z*_*a*/2_)^2^*p*(1 − *p*)/*d*^2^) based on the following assumptions: 95% confidence interval, 5% margin of error and taking 50% magnitude to get maximum sample size on term LBW, and 10% for incompleteness of secondary data (records). Hence, the sample size was 424 live births.

 
(1.96)^2^0.5(1 − 0.5)/(0.05)^2^ = 385 adding 10% for nonresponse rate = 424

Two years mothers' cards were reviewed (37–42 wk of GA). The total mothers who gave live birth neonates were 3000 mothers within the two-year duration (2014–2016). The calculated sample size was 424. The method of sampling we used was systematic random sampling; after we make the complete list of the participants we calculate the interval *K*th = 3000/424 = 7. We select randomly from the first seven listed participants and then we follow the same interval to allocate our sample size. The data extraction form is comprised of variables related to the maternal, neonatal, health related, and sociodemographic characteristics. The form was developed from World Health Organization, Ethiopian Demographic Health Survey, and other literatures. The data was extracted from Adwa General Hospital from two-year list of total term live deliveries obtained from delivery registration. The data was collected using data extraction form. This data collection procedure was carried out by five diploma midwives, and it was supervised by two BSc midwives. The data collector was oriented and trained for one day on how to extract the data, which records need to exclude, and about other data collection process. Brief introduction before and during data collection process was given.

Data quality was managed by recruiting BSc and diploma holders for each data collection and supervising working in the study site (Hospital) based on experience of data collection and training was given for one day on how to collect and supervise the data using the prepared questionnaire. Pretest was done in 42 cards in Ayder Hospital before two weeks of actual data collection which was not included in the study and appropriate modification was made to the questionnaire after analyzing the pretest result like time modification, skipping pattern arrangement, wording, and phrases. Daily supervision, spot checking, and reviewing completed questionnaire was conducted to follow for completeness and consistency of the data (questionnaire) by the supervisors and over all data collection process was controlled by principal investigator.

*Data Management and Analysis*. Data was coded and entered to computer using Epi Data software version 3.1 for its customizing skip benefit and finally data was tranfered to SPSS version 20 software package for analysis purpose. Data exploration was undertaken to see if there are odd codes or items that are not logical and then subsequent editing and cleaning were done before analysis.

Data was analyzed using SPSS version 20.0 computer software package. Data cleaning and editing were carried out. Frequency with percentage, pie charts, and bar graphs were used to represent results of categorical variables while mean (SD) and median (IQR) were used to represent continuous variables. Binary logistic regression (odds ratio and 95 percent confidence interval) was used to see the strength of association between dependent variable and each independent variable. Finally, multivariable logistic regression was used to see the predictors of the outcome variable. Variables with *p* value ≤ 0.25 at binary logistic were further analyzed in the multivariable binary logistic regression. Statistical significance for the association was considered at *p* value < 0.05. Binary and multiple logistic regressions were done to identify factors associated with term low birth weight neonate. Crude odds ratios were estimated for all independent variables in the binary logistic regression. All independent variables with *p* value less than 0.25 at bivariate analysis were entered into multivariate logistic regression to control for all possible confounders.

*Ethical Clearance*. Ethical clearance was obtained from the Institutional Ethical Review Board of Mekelle University, College of Health Sciences. Confidentiality was maintained through anonymity in the use of the questionnaire. Medical card room workers were informed regarding the aim of the study. In order to maintain confidentiality of any information provided by the study subjects ID cards were used. Only the data collector and supervisor were present during the record extraction.

*Operational Definitions*

- Term low birth weight: a baby born after gestational age of 37–42 weeks with birth weight of less than 2,500 grams
- Obstetric health condition: the health condition of the mother during pregnancy and delivery

## 3. Results

### 3.1. Sociodemographic Characteristics of the Mothers

The study involved a total of 424 mothers which is 100% of mother charts reviewed. The median (±IQR) age of the participants was 28 (±13 years); 272 (64%) of them were in the range of 20–34 years. 384 (90.6%) of the mothers were married and 219 (51.7%) of mothers' weights were above the mean. Slightly above half 241 (56.8%) of the study participants were rural residents. The majority of the mothers came from the Tigray community forming 391 (92.2%) and 183 (43.2%) of mothers were Para 2 to 4 (see Table 1).

### 3.2. Obstetric, Medical, and Health-Related Characteristics of the Mothers

Out of 424 mothers cards enrolled in the study 318 (75%) had a history of abortion and 402 (94.9%) wanted the pregnancy. 279 (65.8%) of them had ≥3 live children. Majority of them 384 (90.6%), 385 (90.8%), 397 (93.6%) took iron folic acid, had negative HIV test, and took tetanus toxoid immunization, respectively. 372 (87.7%) of the mothers were nonreactive with VDRL and 218 (51.4%) of the mothers had normal hemoglobin. 289 (44.6%) had less than four ANC follow-ups, and 350 (82.6%) had less than 2-year birth interval. A large number, 352 (87.4%), had no medical illness (see Table 2).

### 3.3. Neonatal History

220 (51.9%) of the neonates were female; 321 (75.7%) of them had poor APGAR SCORES; 377 (88.9%) had no complications after delivery, and 400 (94.3%) had no congenital malformation (see Table 3).

The multivariable analysis of mother's data is shown in Table 4

Age, history of abortion, pregnancy type, hemoglobin status, intake of iron/folic acid, and HIV status were significantly associated with term low birth weight while residence, maternal weight, marital status, parity, tetanus toxoid immunization, family planning, VDRL status, bad obstetric history, sex of neonate, and congenital anomalies were not associated with term low birth weight (see Table 4).

Mothers who delivered at the hospital at the age of less than 20 years were 1.7 more likely to deliver LBW babies than mothers aged 20–34 years (AOR = 1.710, 95% CI: 2.165–17.689). Mothers who wanted the pregnancy were 97% less likely to have LBW babies when compared to those mothers who had unwanted pregnancy (AOR = 0.027, 95% CI: 0.004–0.207). Mothers who had history of abortion were 2.4 times more likely to have LBW babies than those with no history of abortion (AOR = 2.423, 95% CI: 1.744–15.317). Mothers who had normal hemoglobin status were 98% less likely to give birth to LBW babies than those who had abnormal hemoglobin status (AOR = 0.017, 95% CI: 0.002–0.176). Mothers who took iron with folic acid were 99% less likely to have LBW babies than those who did not take iron and folic acid (AOR = 0.007, 95% CI: 0.000–.119). Mothers who were reactive for HIV were seven times more likely to have LBW babies than nonreactive mothers (AOR = 6.121, 95% CI: 1.213–30.897) (Table 4). The prevalence of term LBW was about 10%.

## 4. Discussion

The prevalence of term low birth weight in this study was 10% which is similar to the result in Pakistan, 10.6% [15], but different from the study done in Central India with a prevalence of 33% [16].

Multivariate analysis shows that maternal age, pregnancy type, history of abortion, hemoglobin status, iron folic acid intake, and HIV status were significantly associated with term low birth weight. Also mothers less than 20 years of age delivering at the hospital were most likely to have term low birth weight, which is in agreement with studies done in Nepal [6] and India [9] but contrasts with study done in Gujarat [17]. Teenage pregnancy mostly results in low birth weight. This study shows that history of abortion was significantly associated with term LBW which is consistent with studies from USA [18] and China [19].

The study shows that the type of pregnancy was significantly associated with term low birth weight. Newborns from unwanted and unplanned pregnancies were more likely than those from wanted and planned pregnancies to have LBW. This is similar to study done in Axum [20]. These mothers with unwanted pregnancy may not have proper nutritional status. Other predictor for term low birth weight was normal hemoglobin and shows that anemic mothers were likely to have low birth weight babies compared to nonanemic mothers. This is similar to results of studies from in India [17] and Pakistan [15].

## 5. Conclusion

Prevalence of term low birth weight in this study was almost similar to the results of other studies. The factors associated with term low birth weight are pregnancy type, maternal age history of abortion, hemoglobin status, iron folic acid, and HIV status. The burden of LBW obtained in this study was in the same range as in some other countries.

## Figures and Tables

**Table 1:** Maternal sociodemographic characteristics of the mothers.

Variable	Number	Percent (%)
*Age of mothers *		
<20 years	59	13.9
20–34 years	272	64.2
35–49 years	93	21.9
*Mean weight*		
Below mean	205	48.3
Above mean	219	51.7
Mean maternal weight	50 ± 2.5 kg	
*Marital status*		
Married	384	90.6
Unmarried	40	9.4
*Residence *		
Rural	241	56.8
Urban	183	43.2
*Ethnicity*		
Tigray	391	92.2
Others	33	7.8
*Parity*		
Primipara	181	42.7
2–4 children	183	43.2
≥5 children	60	14.2

**Table 2:** Obstetric, medical, and health-related characteristics of the mothers.

Variable	Number	Percent (%)
History of abortion		
Yes	318	(75.0)
No	106	(25.0)
Alive number of children		
≤2 children	279	(65.8)
≥3 children	145	(34.2)
Birth interval from last child		
≤2 years	350	(82.6)
>2 years	74	(17.4)
Family planning		
Yes	122	(28.8)
No	301	(71.2)
Pregnancy type		
Wanted & planned	402	(94.9)
Unwanted & unplanned	22	(5.1)
ANC status		
Unbooked	60	(14.2)
<4 visits	289	(44.6)
≥4 visits	175	(41.2)
Iron/folic acid intake		
Yes	384	(90.6)
No	40	(9.4)
Tetanus toxoid immunization		
Yes	397	(93.6)
No	27	(6.4)
HIV status		
Nonreactive (−ve)	385	(90.8)
Reactive (+ve)	25	(5.9)
Unknown	14	(3.3)
VDRL		
Nonreactive	372	(87.7)
Reactive	23	(5.5)
Unknown	29	(6.8)
Bad obstetric History		
Yes	144	(34.0)
No	280	(66.1)
Medical illness		
Yes	52	(12.6)
No	352	(87.4)
Hemoglobin status		
Normal	218	(51.4)
Abnormal	206	(48.6)
Degree of severity of anemia		
No anemia	218	(51.4)
Mild	119	(28.1)
Moderate	75	(17.7)
Severe	12	(2.8)

**Table 3:** Neonatal history of the neonates.

Variable	Frequency	Percent (%)
Sex of neonate		
Male	204	(48.1)
Female	220	(51.9)
Complication of neonate		
Yes	47	(11.1)
No	377	(88.9)
Congenital malformations		
Yes	24	(5.7)
No	400	(94.3)
APGAR score		
Poor (<7)	321	(75.7)
Good (≥7)	103	(24.3)

**Table 4:** Multivariable analysis of mothers data from July 1, 2014, to June 30, 2016.

	Term low birth weight		COR (95% CI)	AOD (95% CI)
	No (%)	Yes (%)		
Age of mothers				
<20 years	55 (13.0)	4 (0.9)	0.477 (1.163–10.395)	**1.710 (2.165**–**17.689)**
20–34 years	236 (55.7)	36 (8.5)	1	1
35–49 years	90 (21.2)	3 (0.5)	0.219 (0.066–0.727)	0.0001 (0.000–0.002)^*∗∗*^
Pregnancy type				
Wanted	367 (86.6)	35 (8.3)	5.992 (2.352–15.267)	**0.027 (0.004**–**0.207)**^*∗∗*^
Unwanted	14 (3.3)	8 (1.9)	1	1
History of abortion				
Yes	298 (70.3)	20 (4.7)	0.242 (0.127–0.462)	**2.423 (1.744**–**15.317)**^*∗*^
No	83 (19.6)	23 (5.4)	1	1
Hemoglobin status				
Normal	213 (50.2)	5 (1.2)	9.636 (3.711–25.017)	**0.017 (0.002**–**0.176)**^*∗∗*^
Abnormal	168 (39.6)	38 (9.0)	1	1
Iron and folic acid intake				
Yes	349 (82.3)	35 (8.3)	2.493 (1.066–5.827)	**0.007 (0.002**–**0.119)**^*∗∗∗*^
No	32 (7.5)	8 (1.9)	1	1
HIV status				
Nonreactive	355 (83.7)	30 (7.1)	1	
Reactive	16 (3.8)	9 (2.1)	6.656 (2.713–16.333)	**6.121 (1.213**–**13.897)**^**∗∗**^
Unknown	10 (2.4)	4 (0.9)	4.733 (1.400–16.000)	1.478 (0.191–11.439)

Note ^*∗*^*p* < 0.05, ^*∗∗*^*p* < 0.01, and ^*∗∗∗*^*p* < 0.001.

## References

[1] Deshmukh J. S., Motghare D. D., Zodpey S. P., Wadhva S. K. (1998). Low birth weight and associated maternal factors in an urban area. *Indian Pediatrics*.

[2] Singh G., Chouhan R., Sidhu K. (2009). Maternal factors for low birth weight babies. *Medical Journal Armed Forces India*.

[3] Ezugwu E., Onah H., Ezugwu F., Okafor I. (2009). O282 Low birth weight babies at a tertiary hospital in Enugu, South East Nigeria. *International Journal of Gynecology &amp; Obstetrics*.

[4] Hultman C. M., Torrång A., Tuvblad C., Cnattingius S., Larsson J.-O., Lichtenstein P. (2007). Birth weight and attention-deficit/hyperactivity symptoms in childhood and early adolescence: A prospective Swedish twin study. *Journal of the American Academy of Child and Adolescent Psychiatry*.

[5] Watanabe H. (2008). The effect of prepregnancy body mass index and gestational weight gain on birth weight. *INTECH Open Access Publisher*.

[6] Sharma S. R., Giri S., Timalsina U. (2015). Low birth weight at term and its determinants in a tertiary hospital of nepal: a case-control study. *PLoS ONE*.

[7] Zeleke B. M., Zelalem M., Mohammed N. (2012). Incidence and correlates of low birth weight at a referral hospital in northwest ethiopia. *Pan African Medical Journal*.

[8] Bharati P., Pal M., Bandyopadhyay M., Bhakta A., Chakraborty S., Bharati P. (2011). Prevalence and causes of low birth weight in India. *Malaysian Journal of Nutrition*.

[9] Kahsay Z. (2014). Taddese A low birth weight and associated factors among newborns in Gondar town. *Indo Global Journal of Pharmaticitucal Science*.

[10] Ryan C. A., Ryan F., Keane E., Hegarty H. (2000). Trend analysis and socio-economic differentials in infant mortality in the Southern Health Board, Ireland (1988–1997). *Irish Medical Journal*.

[11] Chiarotti F., Castignani A. M., Puopolo M. (2001). Effects of socio environmental factors on neurocognitive performance in premature or low-birth weight preschoolers. *Ann Ist Super Sanita*.

[12] Aurora S., Vishnu Bhat B., Srinivasan S., Puri R. K., Rajaram P. (1994). Maternal nutrition and birth weight. *Indian J Mat Child Hlth*.

[13] Martin R. J., Fanaroff A. A., Walsh M. C. (2006). Fanaroff and Martin NeonatalPerinatal Medicine, Disease of the fetus and infant.

[14] (2011). U.S. Department of Health and Human Services, Health Resources and Services Administration, Child Health USA 2011. *U.S. Department of Health and Human Services*.

[15] Khan A., Nasrullah F. D., Jaleel R. (2016). Frequency and risk factors of low birth weight in term pregnancy. *Pakistan Journal of Medical Sciences*.

[16] Kumar V. (2010). Magnitude and correlates of low birth weight at term in rural wardha. *Online Journal of Health and Allied Sciences*.

[17] Mumbare S. S., Maindarkar G., Darade R., Yengl S., Tolani M. K., Patole K. (2009). Maternal risk factors associated with term low birth weight neonates: a matched-pair case control study. *Indian Pediatrics*.

[18] Brown J. S., Adera T., Masho S. W. (2008). Previous abortion and the risk of low birth weight and preterm births. *Journal of Epidemiology and Community Health*.

[19] Chen Y., Li G., Ruan Y., Zou L., Wang X., Zhang W. (2013). An epidemiological survey on low birth weight infants in China and analysis of outcomes of full-term low birth weight infants. *BMC Pregnancy and Childbirth*.

[20] Teklehaimanot N., Hailu T., Assefa H. (2009). Prevalence and factors associated with low birth weight in axum and laelay maichew districts, North Ethiopia: a comparative cross sectional study. *International Journal of Nutrition and Food Sciences*.

